# Triterpene Structural Diversification by Plant Cytochrome P450 Enzymes

**DOI:** 10.3389/fpls.2017.01886

**Published:** 2017-11-09

**Authors:** Sumit Ghosh

**Affiliations:** Biotechnology Division, Council of Scientific and Industrial Research-Central Institute of Medicinal and Aromatic Plants, Lucknow, India

**Keywords:** natural product, phytochemical, specialized metabolism, triterpene, sterol, P450

## Abstract

Cytochrome P450 monooxygenases (P450s) represent the largest enzyme family of the plant metabolism. Plants typically devote about 1% of the protein-coding genes for the P450s to execute primary metabolism and also to perform species-specific specialized functions including metabolism of the triterpenes, isoprene-derived 30-carbon compounds. Triterpenes constitute a large and structurally diverse class of natural products with various industrial and pharmaceutical applications. P450-catalyzed structural modification is crucial for the diversification and functionalization of the triterpene scaffolds. In recent times, a remarkable progress has been made in understanding the function of the P450s in plant triterpene metabolism. So far, ∼80 P450s are assigned biochemical functions related to the plant triterpene metabolism. The members of the subfamilies CYP51G, CYP85A, CYP90B-D, CYP710A, CYP724B, and CYP734A are generally conserved across the plant kingdom to take part in plant primary metabolism related to the biosynthesis of essential sterols and steroid hormones. However, the members of the subfamilies CYP51H, CYP71A,D, CYP72A, CYP81Q, CYP87D, CYP88D,L, CYP93E, CYP705A, CYP708A, and CYP716A,C,E,S,U,Y are required for the metabolism of the specialized triterpenes that might perform species-specific functions including chemical defense toward specialized pathogens. Moreover, a recent advancement in high-throughput sequencing of the transcriptomes and genomes has resulted in identification of a large number of candidate P450s from diverse plant species. Assigning biochemical functions to these P450s will be of interest to extend our knowledge on triterpene metabolism in diverse plant species and also for the sustainable production of valuable phytochemicals.

## Introduction

Triterpenes, 30-carbon compounds originated from the 5-carbon isoprene units, constitute a large and structurally diverse class of natural products (Hill and Connolly, 2017). The majority of the triterpene structural diversity is revealed within the plant kingdom. Plants often biosynthesize triterpenes in response to the developmental cues and environmental stimuli (Phillips et al., 2006; Misra et al., 2014; Moses et al., 2015a,b). Although, the actual biological function of most of the plant triterpenes is yet to be revealed, some triterpenes are known for their roles in plant developmental processes and defense response. Anti-microbial triterpene glycosides (avenacins) that accumulate constitutively in roots of the oat plant conferred defense against the root-infecting fungal pathogens (Papadopoulou et al., 1999). However, a few triterpenes were suggested to play crucial function during plant growth and organ development, e.g., lupeol in nodulation (Delis et al., 2011), β-amyrin in nodulation (Confalonieri et al., 2009), and root development (Kemen et al., 2014), thalianol in plant growth and development (Field and Osbourn, 2008), β-amyrin/dihydro-lupeol in root growth and flowering (Krokida et al., 2013), and marneral-derived triterpene(s) in shoot and root development, flowering and embryogenesis (Go et al., 2012). Owing to the potent bioactivities, triterpenes have commercial applications in pharmaceutical, food and cosmetic sectors (Laszczyk, 2009; Sawai and Saito, 2011; Salvador et al., 2012; Moses et al., 2013). Plant-origin triterpenes are being used as dietary supplements and over-the-counter drugs and, moreover, some semi-synthetic triterpene derivatives are undergoing clinical trials (Sheng and Sun, 2011; Moses et al., 2013). For these reasons, triterpene biosynthesis and accumulation processes in plants are studied to a great extent and efforts were also being made to generate alternate sources for the sustainable production of the industrially relevant triterpenes (Moses et al., 2013; Dai et al., 2014; Luo et al., 2015; Arendt et al., 2017; Reed et al., 2017).

2,3-Oxidosqualene is a common biosynthetic precursor for the diverse triterpene skeletons produced in plants. Oxidosqualene cyclases (OSCs) catalyze the first diversifying step for triterpene biosynthesis by converting 2,3-oxidosqualene to a variety of cyclic triterpene scaffolds (Misra et al., 2014; Thimmappa et al., 2014; Ghosh, 2016). In majority of the cases, the cyclic triterpene scaffolds originated from the OSC-catalyzed reactions undergo a plethora of scaffold-, regio-, and stereo-specific oxidations catalyzed by the cytochrome P450 monooxygenases (P450s), leading to triterpene scaffold decoration with various functional groups such as hydroxyl, carbonyl, carboxyl, and epoxy moieties. Moreover, P450-mediated addition of oxygen functionality makes triterpene scaffolds subsequently accessible to the UDP-glycosyltransferases (UGTs) and acyltransferases (ATs) for the generation of the glycosylated (i.e., saponins) and acylated triterpenes (Osbourn et al., 2011; Mugford et al., 2013; Moses et al., 2014a). In recent years, a notable progress has been made in understanding the biochemical functions of the P450s involved in plant triterpene metabolism. Together with the genetic screening of the mutant lines impaired with triterpene biosynthesis, the availability of the genomic and transcriptomic resources led to the identification of a number of P450s that are involved in plant triterpene structural modifications (Qi et al., 2006; Carelli et al., 2011; Augustin et al., 2015; Biazzi et al., 2015; Miettinen et al., 2017; Misra et al., 2017). This article highlights the diverse roles of the plant P450s in triterpene scaffold modifications.

## Biosynthetic Pathway Leading to the Triterpene Structural Diversity

To date, >23,000 triterpene structures are known from the natural sources. These are build-up on >100 structural scaffolds ranging from acyclic to hexacyclic structures (Hill and Connolly, 2017). Among these, tetracyclic and pentacyclic scaffolds represent the major triterpene classes (Ghosh, 2016). Triterpene scaffolds are decorated with various functional groups such as hydroxyl, carbonyl, carboxyl, epoxy, alkyl, acyl, malonyl, and glycosyl leading to a huge structural diversity (Osbourn et al., 2011; Moses et al., 2014a; Thimmappa et al., 2014).

Triterpenes are generally produced from the acyclic 30-carbon precursors squalene in bacteria and 2,3-oxidosqualene in eukaryotes. However, a few exceptions were also known. Green algae produce botryococcene and their methylated derivatives from farnesyl pyrophosphate (Jiang et al., 2016). Moreover, some bacteria use 2,3-oxidosqualene as biosynthetic precursor for the sterol (Bode et al., 2003; Wei et al., 2016). In eukaryotes, 2,3-oxidosqualene also serves a common precursor for the biosynthesis of the sterols and steroid hormones that play primary function during growth and development of the organisms (Benveniste, 2004).

The conversion of 2,3-oxidosqualene into diverse cyclic triterpenes by OSCs, the class II terpene synthases is the first diversifying step of the triterpene biosynthetic pathway, and also marks the branch point for the biosynthesis of the sterols and steroid hormones (Thimmappa et al., 2014; Ghosh, 2016). Unlike animals and fungi genomes that generally encode a single OSC (lanosterol synthase) for membrane sterol and steroid hormone biosynthesis, the higher plant genomes encode several OSCs (e.g., 9 and 13 in rice and Arabidopsis, respectively) for the biosynthesis of the sterol (cycloartenol synthase and lanosterol synthase) and triterpene (e.g., β-amyrin synthase) scaffolds (Sawai and Saito, 2011; Thimmappa et al., 2014; Ghosh, 2016). So far, about 100 OSCs are identified from the plants and biochemically characterized to reveal their product specificities. More than two-third of these OSCs were found to have product specificity for single triterpene (mono-functional OSCs). Other OSCs converted 2,3-oxidosqualene into triterpene products, ranging from 2 to 23 in number (multi-functional OSCs). Subsequent of the OSC-mediated cyclization, triterpene and sterol scaffolds undergo a plethora of structural modifications catalyzed by the P450s, ATs, and UGTs leading to scaffold-, regio-, and stereo-specific oxidation, methylation, acetylation, malonylation, and glycosylation (Osbourn et al., 2011). The reactions catalyzed by the P450s were found to be extremely diverse in nature, including oxidation, desaturation, and C–C bond cleavage. P450-catalyzed reactions of the plant triterpene pathways are discussed in the following sections.

## P450 Nomenclature and Classification

**FIGURE 1:**
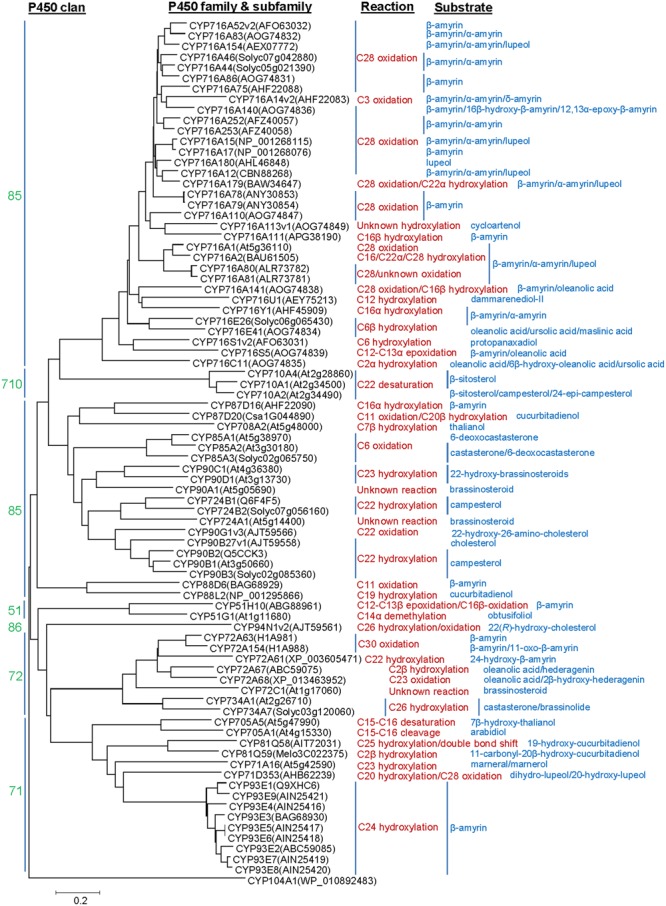
Phylogenetic analysis of the P450s known to catalyze structural modifications on steroidal and triterpene scaffolds. Complete amino acid sequences of the P450s were analyzed by MEGA6. Sequence alignment was carried out following ClustalW analysis and phylogenetic tree was built using the Neighbor Joining Method. Evolutionary distances were computed using the Poisson correction method and are in the units of the number of amino acid substitutions per site. *Agrobacterium tumefaciens* CYP104A1 was included as out group.

P450s representing about 1% of the protein coding genes of the plant genomes, constitute the largest family of the enzymes involved in plant metabolism (Nelson and Werck-Reichhart, 2011). P450s are generally classified into different families and subfamilies based on sequence homology and phylogenetic criteria. The nomenclature for the P450s is provided in a chronological order based on sequence submission to the P450 Nomenclature Committee (David Nelson: dnelson@uthsc.edu). P450s having >40% and >55% amino acid sequence homology are assigned a same family and subfamily, respectively. So far, 127 P450 families are known from the plants as compared to 19, 67, 333, and 399 families in vertebrates, insects, bacteria, and fungi, respectively (Nelson, 2011). The land plant P450 families were categorized into 11 clans that represented distinct clades on a phylogenetic tree (Nelson and Werck-Reichhart, 2011). The clans CYP51, CYP74, CYP97, CYP710, CYP711, CYP727, and CYP746 included single P450 family; however, the clans CYP71, CYP72, CYP85, and CYP86 represented multiple P450 families. So far, the clans CYP51 (member: CYP51H; 51 and H denote family and subfamily, respectively), CYP71 (members: CYP71A, CYP71D, CYP81Q, CYP93E, and CYP705A), CYP72 (member: CYP72A) and CYP85 (members: CYP87D, CYP88D, CYP88L, CYP708A, CYP716A, CYP716C, CYP716E, CYP716S, CYP716U, and CYP716Y) were associated with the triterpene structural modifications (**Figure 1**). Besides the clans CYP51 (member: CYP51G) and CYP85 (members: CYP85A, CYP90B, CYP90C, CYP90D, CYP90G, CYP716A, CYP724A, and CYP724B), the clans CYP72 (member: CYP734A), CYP86 (member: CYP94N) and CYP710 (member: CYP710A) were known for the sterol scaffold modifications (**Figure 1**).

## P450 In Modification of the Sterol Scaffolds

Phytosterols (e.g., campesterol, β-sitosterol, stigmasterol, and brassicasterol), steroid hormones (e.g., brassinosteroids), steroidal alkaloids (e.g., cyclopamine, chaconine, solanine, and tomatine), and steroidal saponins (e.g., asparasaponins, withalongolides, and avenacosides) are all derived from the cycloartenol scaffold. P450s involved in modification of the sterol scaffolds are discussed in the following sections.

### CYP51G-Sterol 14α-Demethylase

**FIGURE 2:**
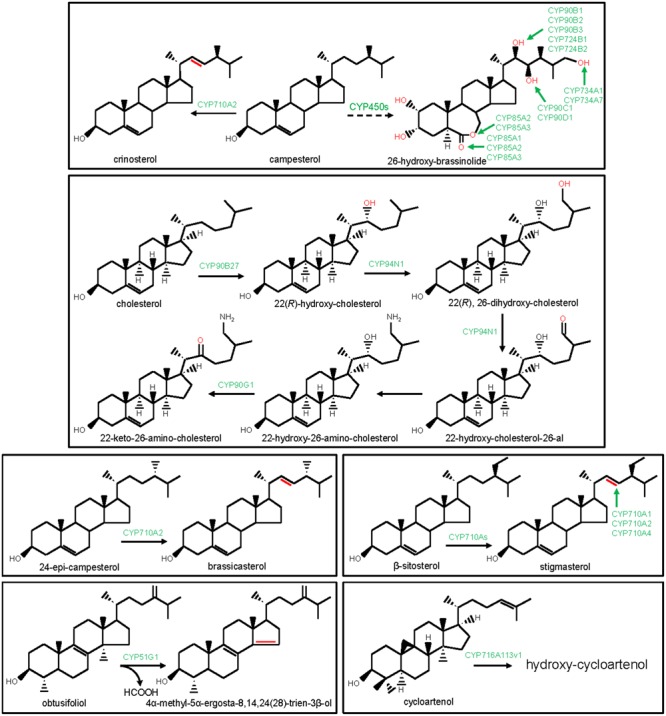
Modification of the steroidal scaffolds by the P450s. The structural modifications catalyzed by the plant P450s are indicated with red color. P450s are highlighted with green color.

An initial reaction of the phytosterol biosynthesis is the CYP51G-catalyzed 14α-demethylation of obtusifoliol (Bak et al., 1997; Kim et al., 2005). CYP51G catalyzes the conversion of 14α-methyl group of obtusifoliol into 14α-carboxyaldehyde, involving two consecutive oxidation reactions and finally, the elimination of the 14α-aldehyde group as formic acid with concomitant formation of Δ14,15 double bond into the sterol scaffold (Shyadehi et al., 1996; Bak et al., 1997; Kim et al., 2005; Waterman and Lepesheva, 2005; **Figure 2**). CYP51 function remained conserved in fungi and animals as 14α-demethylase of sterol precursors such as lanosterol, dihydrolanosterol, and eburicol. In animals and fungi, CYP51 members are designated as CYP51A and ERG11/CYP51F, respectively. Moreover, protozoan and bacterial genomes also encoded sterol 14α-demethylase, designated as CYP51E and CYP51B, respectively. The bacterial genomes possibly gained CYP51 members through horizontal transfer from the eukaryotic genomes (Rezen et al., 2004). The biochemical and phenotypic analysis of the Arabidopsis CYP51G1 loss-of-function mutants established an essential role of the CYP51G1 in plant growth and development (Kim et al., 2005). Similarly, CYP51A and ERG11 functions are essential for the survival of the animals and fungi, respectively (Bard et al., 1993). CYP51 is the primary target of the anti-fungal (azoles) drugs and agricultural fungicides. Moreover, CYP51 is also a potential target for the development of anti-trypanosomal chemotherapy.

### CYP710A-Sterol 22 Desaturase

CYP710A participates in sterol biosynthesis by catalyzing sterol C22 desaturation reaction. Among the four CYP710A members of Arabidopsis, CYP710A1, CYP710A2, and CYP710A4 were biochemically characterized (Morikawa et al., 2006; Arnqvist et al., 2008). In *in vitro* assays, CYP710A1, CYP710A2, and CYP710A4 catalyzed the conversion of β-sitosterol to stigmasterol (**Figure 2**). Moreover, CYP710A2 also converted campesterol to crinosterol and 24-epi-campesterol to brassicasterol (**Figure 2**). However, based on gene over-expression studies in Arabidopsis, the *in planta* functions of CYP710A1 and CYP710A4 in stigmasterol biosynthesis, and CYP710A2 in brassicasterol and crinosterol biosynthesis were established (Morikawa et al., 2006; Arnqvist et al., 2008). Further substantiating these observations, *cyp710a2* null mutant did not accumulate brassicasterol and crinosterol (Morikawa et al., 2006). Similar to the higher plants, *Physcomitrella patens* also engaged CYP710A for sterol C22 desaturation, signifying evolutionary conservation of the CYP710A’s function in sterol biosynthesis (Morikawa et al., 2009).

### P450 in Brassinosteroids Biosynthesis

The biosynthesis and catabolism of brassinosteroids (BRs), the poly-hydroxylated steroid hormones that mediate plant growth and development, involve P450s-catalyzed regio- and stereo-specific oxidation of the campesterol scaffold at the C2, C3, C6, C22, C23, and C26 positions. The P450 family members (CYP72C, CYP85A, CYP90B-D, CYP724A,B, and CYP734A) that catalyze the consecutive biochemical reactions of the BRs pathway, were identified (**Figure 2**). The genetic screening of the mutant lines and biochemical analysis of the corresponding enzymes established the role of the CYP90B and CYP724B subfamily members in catalyzing the conversion of campesterol to 22-hydroxy-campesterol in Arabidopsis, rice, and tomato (Choe et al., 2001; Ohnishi et al., 2006c; Sakamoto et al., 2006). Likewise, Arabidopsis CYP90C1 and CYP90D1 have been implicated in BRs biosynthesis pathway as steroid C23-hydroxylases (Ohnishi et al., 2006b). Besides, Arabidopsis and tomato CYP85A subfamily members were known for the C6 oxidation activities in BR biosynthesis. Arabidopsis CYP85A1 and tomato ortholog catalyzed C6 oxidation of 6-deoxocastasterone leading to castasterone. However, Arabidopsis CYP85A2 and tomato CYP85A3 were responsible for the C6 oxidation of 6-deoxocastasterone leading to castasterone as well as the conversion of castasterone to brassinolide by Baeyer-Villiger oxidation (Shimada et al., 2001; Nomura et al., 2005). Arabidopsis CYP90A1 and CYP724A1 were also associated with the BRs biosynthetic pathway; however, their exact biochemical functions remain to be clarified (Szekeres et al., 1996; Bak et al., 2011). Moreover, genetic screening of the Arabidopsis activation tagged lines revealed the role of the CYP72C1 and CYP734A1 in BRs catabolism (Neff et al., 1999; Takahashi et al., 2005). CYP734A1 catalyzed C26 hydroxylation of bioactive brassinosteroids such as castasterone and brassinolide. However, the biochemical function of the CYP72C1 is yet to be known (Turk et al., 2003). Unlike Arabidopsis-specific CYP72C1, the function of the CYP734A1 appears to be conserved in other plants, as revealed for the tomato ortholog CYP734A7 (Ohnishi et al., 2006a).

### P450 in Steroidal Alkaloid and Steroidal Saponin Biosynthesis

The biosynthetic enzymes for the cyclopamine, a steroidal alkaloid that showed promising anti-neoplastic activities, were identified from *Veratrum californicum* (Lin et al., 2010; Augustin et al., 2015). These enzymes included three P450 family members (CYP90B27, CYP90G1, and CYP94N1) that were recently identified by exploring transcriptome (RNA-seq) data of *V. californicum* (**Figure 2**). These P450s were biochemically characterized by employing a protein expression platform based on Sf9 insect cells (Augustin et al., 2015). CYP90B27 being a cholesterol 22-hydroxylase, catalyzed the conversion of cholesterol to 22(R)-hydroxy-cholesterol. However, CYP94N1 converted 22(R)-hydroxy-cholesterol to 22-hydroxy-cholesterol-26-al following sequential two-step oxidations at the C26 position. CYP90G1 was designated as 22-hydroxy-26-amino-cholesterol 22-oxidase that converted 22-hydroxy-26-amino-cholesterol to 22-keto-26-amino-cholesterol. Similar to CYP90G1, CYP90B27 was also found to oxidize C22 hydroxyl to C22 ketone, however, only with a lower efficiency than the CYP90G1 (Augustin et al., 2015).

Recently, a member of the CYP716A subfamily (CYP716A113v1) was identified from the basal eudicot *Aquilegia coerulea* and suggested to take part in steroidal saponin biosynthesis (Miettinen et al., 2017). CYP716A113v1 was found to hydroxylate the steroidal saponin precursor cycloartenol; however, the exact regio- and stereo-chemistry of the oxidation reaction remain to be known.

## P450 In Pentacyclic Triterpene Biosynthesis

Oleanane, ursane, and lupane, derived from β-amyrin, α-amyrin, and lupeol, respectively, represent the major pentacyclic triterpene scaffolds (Ghosh, 2016). To date, about 55 P450s are identified that act on plant pentacyclic triterpene scaffolds. The majority of them belong to the CYP716 family members. CYP51, CYP71, CYP72, CYP87, CYP88, and CYP93 are the other P450 families that were also found to participate in pentacyclic triterpene modifications (**Figure 1**). P450 functions in mediating structural diversification of the pentacyclic triterpenes are discussed in the following sections.

### Eudicot CYP716A, CYP716C, CYP716E, CYP716S, and CYP716Y in Pentacyclic Triterpene Biosynthesis

**FIGURE 3:**
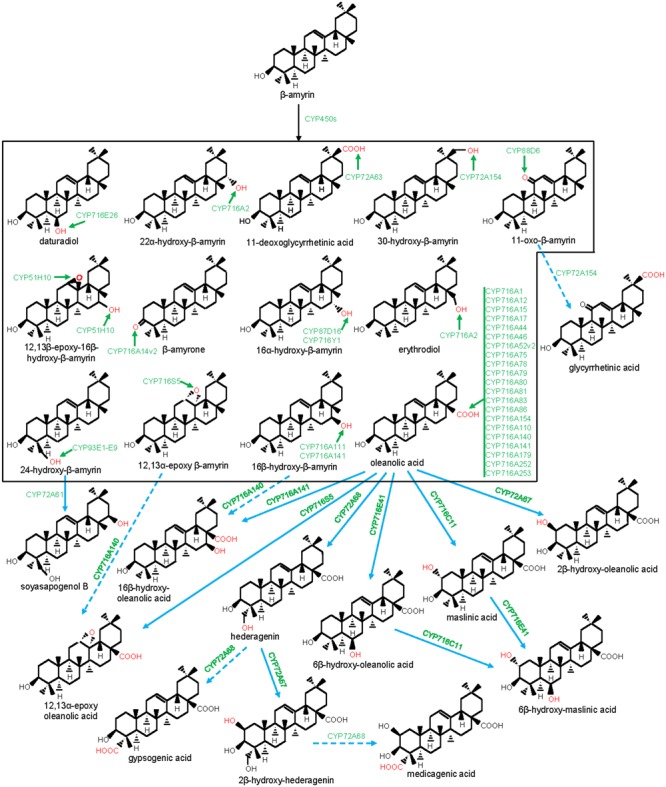
Modification of the oleanane-type triterpene scaffolds by the P450s. The structural modifications catalyzed by the plant P450s are indicated with red color. P450s are highlighted with green color. Dashed arrow indicates that the reaction is processed through intermediate compound(s).

**FIGURE 4:**
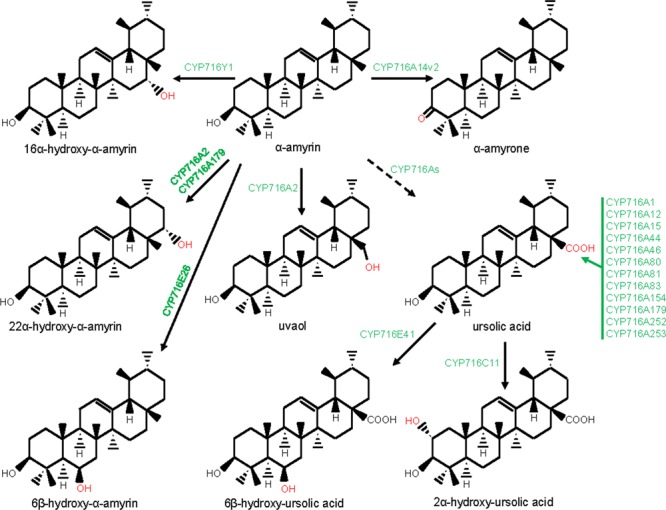
Modification of the ursane-type triterpene scaffolds by the P450s. The structural modifications catalyzed by the plant P450s are indicated with red color. P450s are highlighted with green color. Dashed arrow indicates that the reaction is processed through intermediate compound(s).

**FIGURE 5:**
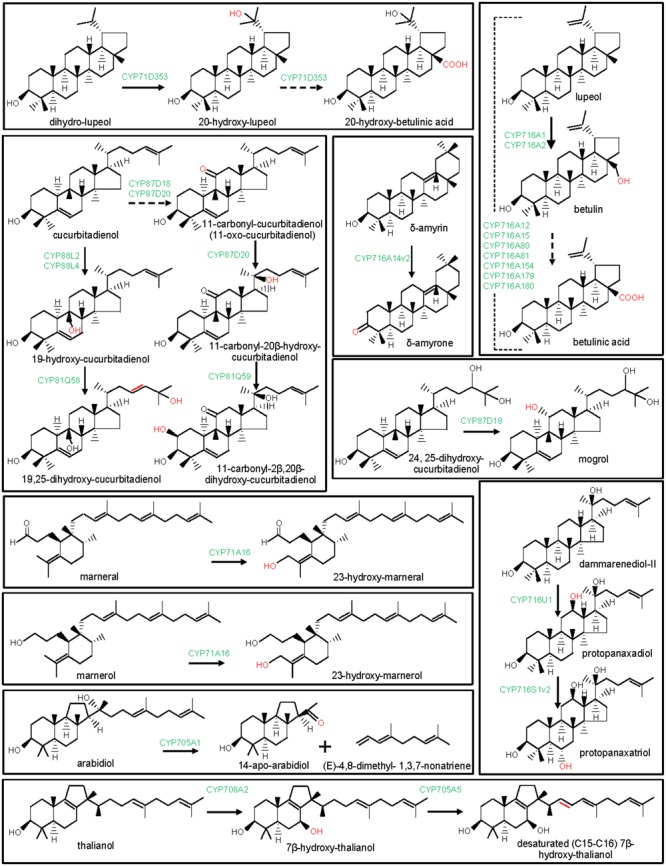
Modification of the lupane, dammarane, cucurbitane, and other triterpene scaffolds by P450s. The structural modifications catalyzed by the plant P450s are indicated with red color. P450s are highlighted with green color. Dashed arrow indicates that the reaction is processed through intermediate compound(s).

CYP716s are classified under the clan CYP85. They were evolved early with the land plants and were found in the genomes of the bryophytes, lycopods, ferns, gymnosperms, and angiosperms, however, not in monocot (Nelson and Werck-Reichhart, 2011). Although, the function of the lower plant CYP716As is yet to be clarified, the majority of the eudicot CYP716s was found to participate in pentacyclic triterpene scaffold modifications. A recent report of kingdom-wide phylogenetic analysis of CYP716s, collected from >200 plant species indicated that, in eudicots, CYP716 family evolved specifically toward triterpene biosynthesis (Miettinen et al., 2017). The first evidence for the CYP716s participation in triterpene biosynthesis appeared from a gene co-expression study in Arabidopsis that suggested a co-regulated expression of *CYP716A1* and *CYP716A2* with *PEN3*, an OSC (Ehlting et al., 2008). However, for the first time, the genetic and biochemical evidences were gathered following characterization of a *Medicago truncatula* mutant line deficient in hemolytic saponin biosynthesis with a lesion in *CYP716A12* gene (Carelli et al., 2011). It was found that CYP716A12 partakes in *M. truncatula* hemolytic saponin biosynthesis by catalyzing the sequential three-step oxidation at the C28 position of β-amyrin, leading to the formation of oleanolic acid (Carelli et al., 2011; Fukushima et al., 2011). Besides, *in vitro* and *in vivo* assays using heterologous system also revealed the ability of the CYP716A12 in converting α-amyrin to ursolic acid and lupeol to betulinic acid, following three-step oxidation at the C28 position (Carelli et al., 2011; Fukushima et al., 2011). Subsequent to these initial reports, several CYP716s were identified and biochemically characterized from the plants, mostly by exploring transcriptomic and genomic resources (Misra et al., 2017; Miettinen et al., 2017; Tamura et al., 2017a). To date, about 20 CYP716As are biochemically characterized for the amyrin/lupeol C28-oxidase activities (**Figures 1, 3–5**). A majority of these CYP716As catalyzed sequential three-step oxidation of the amyrin/lupeol scaffolds, leading to the consecutive formation of the hydroxyl, aldehyde, and carboxyl moieties at the C28 position (Carelli et al., 2011; Fukushima et al., 2011; Misra et al., 2017; Tamura et al., 2017a). A few plant CYP716As also target a carbon atom of the amyrin skeletons other than the C28. These are Arabidopsis CYP716A2 for C22α hydroxylation, *Artemisia annua* CYP716A14v2 for C3 oxidation, and *Aquilegia coerulea* CYP716A111 and *Platycodon grandiflorus* CYP716A141 for C16β hydroxylation (Moses et al., 2015b; Yasumoto et al., 2016; Miettinen et al., 2017; Tamura et al., 2017b).

Besides CYP716A subfamily members, CYP716C, CYP716E, CYP716S, and CYP716Y subfamily members were also known to oxidize pentacyclic triterpene scaffolds (**Figures 1, 3–5**). Tomato CYP716E26 and *Centella asiatica* CYP716E41 were biochemically characterized as C6β-hydroxylases that accepted α/β-amyrin and oleanolic acid/ursolic acid/maslinic acid as substrates, respectively (Miettinen et al., 2017; Yasumoto et al., 2017). Though CYP716E41 did not oxidize amyrin skeleton (Miettinen et al., 2017), whether CYP716E26 can oxidize oleanolic acid/ursolic acid/maslinic acid remains to be tested. *C. asiatica* CYP716C11 was found to catalyze C2α hydroxylation of oleanolic acid, 6β-hydroxy-oleanolic acid, and ursolic acid (Miettinen et al., 2017). However, *P. grandiflorus* CYP716S5 catalyzed C12-C13α epoxidation of β-amyrin and oleanolic acid. Moreover, *Bupleurum falcatum* CYP716Y1 is a C16α-hydroxylase that acted on β-amyrin and α-amyrin scaffolds (Moses et al., 2014b; Miettinen et al., 2017).

### Monocot CYP51H in Pentacyclic Triterpene Biosynthesis

The CYP51 family is conserved across algae to higher plants (Nelson and Werck-Reichhart, 2011). Unlike the conserved function of the CYP51G subfamily members as sterol 14α-demethylase, CYP51H subfamily appears to be specifically recruited for the pentacyclic triterpene scaffold modification in monocots. The CYP51H subfamily members seem to be restricted to the monocots such as oats and rice (Qi et al., 2006). Although the oat CYP51H10 was biochemically and functionally characterized, rice counterpart is yet to be analyzed (Geisler et al., 2013). It might be hypothesized that CYP51H emerged in monocots to compensate the loss of the CYP716 family from monocots in maintaining triterpene structural diversity.

In a forward genetic screen for the avenacin-deficient oat mutants (*sad* mutants), CYP51H10 (*sad2*) was found to be dispensable for essential sterols biosynthesis. However, CYP51H10 was indispensable for the production of anti-microbial oleanane-triterpene saponins (avenacins) that conferred disease resistance in oats toward the root-infecting fungal pathogens (Qi et al., 2006). CYP51H10 catalyzed both C12-C13β epoxidation of the C ring and C16β hydroxylation of the D ring of β-amyrin leading to the formation of 12,13β-epoxy-16β-hydroxy-β-amyrin, an intermediate of the avenacin biosynthetic pathway (**Figure 3**; Kunii et al., 2012; Geisler et al., 2013). Moreover, molecular modeling and docking studies suggested that C16 hydroxylation of the D-ring is likely followed by C12-C13 epoxidation of the C-ring (Geisler et al., 2013).

### Legume CYP72A, CYP88D, and CYP93E in Pentacyclic Triterpene Biosynthesis

Legumes accumulate hemolytic and non-hemolytic triterpene saponins (Moses et al., 2014c; Ghosh, 2016). Hederagenin, zanhic acid, and medicagenic acid scaffolds-derived saponins with oxidation at the C28 position are classified as hemolytic; however, soyasapogenols-derived saponins with oxidation at the C24 position are non-hemolytic (Carelli et al., 2011). In addition, hemolytic saponins also bear oxygen functionality at the C2 and C23 positions as compared to oxygen functionality at the C22 position in non-hemolytic saponins. Besides, CYP716A12 with β-amyrin C28-oxidase activity, CYP72A, CYP88D, and CYP93E subfamily members were known for the biosynthesis of the triterpene saponins in legumes (Seki et al., 2008; Moses et al., 2014c; Biazzi et al., 2015).

The members of the CYP93 family comprising of five subfamilies (A–E) were found in several plant genomes (Nelson and Werck-Reichhart, 2011). The majority of the CYP93 family members were associated with the flavonoid metabolism (Ayabe and Akashi, 2006). However, CYP93E subfamily members appear to be restricted to the legumes for the triterpene saponin biosynthesis (Moses et al., 2014c). The first characterized member of this subfamily is the *Glycine max* CYP93E1 (Shibuya et al., 2006). This enzyme is a C24-hydroxylase that converted β-amyrin and sophoradiol to 24-hydroxy-β-amyrin and soyasapogenol B, respectively, the intermediates for the biosynthesis of the legume-specific soyasaponins of non-hemolytic class. CYP93E1 also represents the first characterized P450 of the triterpene biosynthetic pathway. To date, eight additional CYP93Es (E2-E9) were identified from eight legume species; all of them showed C24-hydroxylase activity (**Figure 3**), suggesting the conserved role of the CYP93E in legume-specific triterpene saponin biosynthesis (Seki et al., 2008; Fukushima et al., 2013; Moses et al., 2014c). Despite the high degree of amino acid conservation (>80% identity), CYP93Es of different legumes exhibited a large variation in β-amyrin C24 hydroxylation efficiency in yeast (*Saccharomyces cerevisiae*). The highest activity was obtained for the *Phaseolus vulgari*s CYP93E9 that showed 61-fold higher activity than the *Medicago truncatula* CYP93E2 (Moses et al., 2014c). However, whether this large variation in C24 hydroxylation efficiency was due to the differential protein expression in heterologous host or the actual difference in the catalytic efficiency of the CYP93Es needs to be tested.

CYP72A subfamily members are distributed well across the angiosperm species and were not found in gymnosperm, moss, and fern species (Prall et al., 2016). However, the biochemical function of most of the angiosperm CYP72As remains to be known. To date, CYP72As of *M. truncatula* and *Glycyrrhiza uralensis* were biochemically characterized and were found to be associated with the legume-specific triterpene saponin biosynthesis. So far, four CYP72As of *M. truncatula* (CYP72A61, CYP72A63, CYP72A67, and CYP72A68) and one CYP72A of *G. uralensis* (CYP72A154) were reported to partake in oleanane triterpene scaffold oxidation (**Figures 1, 3**; Seki et al., 2011; Fukushima et al., 2013; Biazzi et al., 2015). *M. truncatula* CYP72A61, a C22-hydroxylase of the non-hemolytic saponin biosynthesis pathway, converted 24-hydroxy-β-amyrin (the product of the CYP93E) to soyasapogenol B. However, CYP72A67 and CYP72A68 were found to be C2β-hydroxylase (oleanolic acid/hederagenin C2β-hydroxylase) and C23-oxidase (oleanolic acid/hederagenin/2β-hydroxy-hederagenin C23-oxidase) of the hemolytic saponin pathway, respectively (Fukushima et al., 2013; Biazzi et al., 2015). Conversely, *G. uralensis* CYP72A154 is a C30-oxidase that catalyzed three-step oxidation of 11-oxo-β-amyrin to glycyrrhetinic acid, an intermediate for the biosynthesis of glycyrrhizin (Seki et al., 2011). Glycyrrhizin, a triterpene saponin derived from the underground parts of the *Glycyrrhiza* (licorice) plants has various pharmaceutical properties and is being used worldwide as a natural sweetener (Seki et al., 2008). CYP72A154 also reacted on β-amyrin and converted it to 30-hydroxy-β-amyrin. Similarly, *M. truncatula* CYP72A63 was found to possess C30-oxidase activity and converted β-amyrin to 11-deoxoglycyrrhetinic acid following a three-step oxidation (Seki et al., 2011). Besides legumes, other characterized CYP72As are from *Catharanthus roseus* (CYP72A1 and CYP72A224) that participate in indole alkaloid biosynthesis (Irmler et al., 2000; Salim et al., 2013; Miettinen et al., 2014).

CYP88D6 is another enzyme of the *G. uralensis* glycyrrhizin biosynthesis pathway (Seki et al., 2008). CYP88D6 catalyzed sequential two-step oxidation of β-amyrin at C11 to produce 11-oxo-β-amyrin, the substrate for CYP72A154. Other characterized members of this family (CYP88A3 and CYP88A4) have been shown to be involved in the biosynthesis of the plant hormone gibberellins (Helliwell et al., 2001).

### CYP71D and CYP87D in Pentacyclic Triterpene Biosynthesis

The clan CYP71 represents the largest set of the plant P450s. The families and subfamilies within the clan CYP71 diverged remarkably during plant evolution (Nelson et al., 2004; Nelson and Werck-Reichhart, 2011). Beside CYP93E subfamily, CYP71A, CYP71D, CYP81Q, and CYP705A subfamilies of the clan CYP71 were also found to take part in plant triterpene metabolism (**Figure 1**). Among these, *Lotus japonicus* CYP71D353 had been shown to oxidize lupane-type pentacyclic triterpene scaffold (Krokida et al., 2013). CYP71D353 catalyzed conversion of dihydro-lupeol to 20-hydroxy-lupeol following hydroxylation at the C20 position. CYP71D353 also converted 20-hydroxy-lupeol to 20-hydroxy-betulinic acid in a sequential three-step oxidation at the C28 position (**Figure 5**). CYP71D353 still remains the only known member of the CYP71D subfamily with a role in triterpene biosynthesis. Some other CYP71D subfamily members were known to take part in monoterpene and flavonoid hydroxylation (Haudenschild et al., 2000; Latunde-Dada et al., 2001).

Besides CYP88D and CYP716s, CYP87D is another subfamily under the CYP85 clan with a role in pentacyclic triterpene scaffold modification (**Figure 1**). To date, CYP87D16 of *Maesa lanceolata* is the only known member of the CYP87D subfamily associated with the pentacyclic triterpene scaffold modification (Moses et al., 2015a). CYP87D16 participates in triterpene saponin pathway by catalyzing C16α hydroxylation of β-amyrin. The same biochemical activity was also reported for the *B. falcatum* CYP716Y1 that showed only 27% homology with the CYP87D16 at the protein level (Moses et al., 2014b). So far, CYP87D16 and CYP716Y1 represent the only known examples of the P450s that belong to the separate P450 families and, however, possess the same biochemical function, suggesting that C16α hydroxylation activity might have evolved independently in different plant species.

## P450 In Tetracyclic Triterpene Biosynthesis

Tetracyclic scaffolds, e.g., lanostane, cucurbitane, protostane, dammarane, euphane, tirucallane constitute a major triterpene class (Ghosh, 2016). So far, P450s that catalyzed modifications of the cucurbitane and dammarane scaffolds are known (**Figures 1, 5**). These are CYP716A47 (renamed as CYP716U1) and CYP716A53v2 (renamed as CYP716S1v2) of *Panax ginseng*, CYP87D18 of *Siraitia grosvenorii*, CYP81Q58 and CYP88L2 of *Cucumis sativus*, CYP87D20 of *C. sativus, C. melo*, and *Citrullus lanatus* and CYP81Q59 of *C. melo* and *C. lanatus* (Han et al., 2011, 2012; Shang et al., 2014; Zhang et al., 2016; Zhou et al., 2016). *Panax ginseng* CYP716U1 and CYP716S1v2 catalyzed two consecutive hydroxylation reactions in the biosynthetic pathway for the ginsenosides, pharmacologically active dammarane-type triterpene glycosides (Han et al., 2011, 2012). CYP716U1 was found to be involved in the hydroxylation of dammarenediol-II at the C12 position, yielding protopanaxadiol. However, CYP716S1v2 catalyzed hydroxylation at the C6 position of protopanaxadiol to produce protopanaxatriol.

*Cucumis sativus* CYP88L2 and CYP81Q58 catalyzed two successive oxidation reactions in the biosynthetic pathway for the cucurbitacins (**Figure 5**; Shang et al., 2014). Moreover, CYP87D20 is a multifunctional oxidase of the cucurbitacin biosynthetic pathway (**Figure 5**; Zhou et al., 2016). Cucurbitacins are the bitter-tasting cucurbitane-type triterpenes that provide protection to the pests and have also been shown to possess antitumor and hepatoprotective properties (Balkema-Boomstra et al., 2003; Thoennissen et al., 2009). The biochemical function of CYP88L2 was established as a C19-hydroxylase that converted cucurbitadienol to 19-hydroxy-cucurbitadienol. However, CYP81Q58 catalyzed the C25 hydroxylation and a C–C double bond shift (C24,C25 to C23,C24) in 19-hydroxy-cucurbitadienol, leading to the generation of 19,25-dihydroxy-cucurbitadienol (Shang et al., 2014). The multifunctional CYP87D20 catalyzes consecutive C11 oxidation and C20 hydroxylation of cucurbitadienol, leading to the formation of 11-carbonyl-20β-hydroxy-cucurbitadienol (Zhou et al., 2016). Furthermore, a C2β-hydroxylase (CYP81Q59) that converted 11-carbonyl-20β-hydroxy-cucurbitadienol to 11-carbonyl-2β,20β-dihydroxy-cucurbitadienol was known in *C. melo* and *C. lanatus* (Zhou et al., 2016). CYP81Q58 and CYP87D20 activities appear to be required for the common structural modifications in cucurbitacin B, cucurbitacin C, cucurbitacin E in *C. melo, C. sativus*, and *C. lanatus*, respectively. Although, CYP81Q58 of *C. sativus* and CYP87D20 orthologs in *C. sativus, C. melo*, and *C. sativus* were biochemically characterized, CYP81Q58 orthologs in *C. melo* and *C. lanatus* are yet to be studied (Shang et al., 2014; Zhou et al., 2016). However, C19 hydroxylation by CYP88L2 is specific to the *C. sativus* for the biosynthesis of the cucurbitacin C, whereas C2β hydroxylation by CYP81Q59 is restricted to the *C. melo* and *C. lanatus* for the biosynthesis of the cucurbitacin B and cucurbitacin E, respectively (Zhou et al., 2016). CYP88L2 orthologs were either missing or truncated in the *C. melo* and *C. lanatus* genomes, whereas CYP81Q59 transcript was not expressed in *C. sativus* (Zhou et al., 2016). Thus, CYP81Q59 and CYP88L2 are selectively recruited to produce structurally diverse cucurbitacins in different species of the cucurbitaceae family.

*Siraitia grosvenorii*, another member of the cucurbitaceae family, also biosynthesizes cucurbitane-type triterpenes mogrosides which are, however, glycosylated and have sweet taste. Mogrosides exhibited notable pharmaceutical properties and are commercially utilized as sweeteners (Xia et al., 2008). *In vitro* enzymatic activity assays and functional expression in yeast suggested that CYP87D18 encodes a C11-oxidase that catalyzed sequential two-step oxidative reactions at C11 of cucurbitadienol to produce 11-hydroxy-cucurbitadienol and 11-oxo-cucurbitadienol (Itkin et al., 2016; Zhang et al., 2016). CYP87D18 also converted 24,25-epoxy-cucurbitadienol to 11-oxo-24,25-epoxy-cucurbitadienol (Zhang et al., 2016). Moreover, the expression of CYP87D18 in engineered yeast strain that accumulated 24,25-dihydroxy-cucurbitadienol led to the formation of 11,24,25-trihydroxy-cucurbitadienol (mogrol), an aglycone of mogrosides, suggesting CYP87D18-catalyzed C11 hydroxylation of 24,25-dihydroxy-cucurbitadienol (Itkin et al., 2016). Furthermore, protein-modeling and docking studies substantiated the most likely involvement of CYP87D18 in C11 hydroxylation of 24,25-dihydroxy-cucurbitadienol in the mogrosides biosynthetic pathway (Itkin et al., 2016).

## P450 In Biosynthesis of Other Triterpene Classes

A few P450s of Arabidopsis were identified and biochemically characterized for their involvement in monocyclic (marnerol and marneral) and tricyclic (arabidiol and thalianol) triterpenes metabolism (**Figures 1, 5**). Arabidopsis CYP71A16 was found to hydroxylate both marnerol and marneral at the C23 position, when expressed in yeast along with an OSC, marneral synthase (MRN1) (Field et al., 2011; Castillo et al., 2013). Moreover, the *in planta* role of CYP71A16 in triterpene oxidation was also confirmed following analysis of the Arabidopsis null mutants (*mro1-1* and *mro1-2*) and over-expression lines (Field et al., 2011). CYP71A16 co-expressed and clustered in Arabidopsis genome with the MRN1. Based on similar experimental approach, the roles of Arabidopsis CYP708A2 and CYP705A5 in hydroxylation of thalianol to 7β-hydroxy-thalianol and in desaturation of 7β-hydroxy-thalianol, respectively, were also reported (Field and Osbourn, 2008; Castillo et al., 2013). CYP708A2 and CYP705A5 catalyzed two consecutive reactions of the thalianol pathway (**Figure 5**). These genes co-expressed and clustered in the Arabidopsis genome with the OSC thalianol synthase (Field and Osbourn, 2008).

Arabidopsis CYP705A1 that co-expressed and clustered with the OSC arabidiol synthase, catalyzed an unusual C-C bond cleavage reaction (Castillo et al., 2013; Sohrabi et al., 2015). CYP705A1 cleaved tricyclic triterpene arabidiol to a C19 ketone derivative (14-apo-arabidiol) and a C11 product (E)-4,8-dimethyl- 1,3,7-nonatriene (DMNT) (**Figure 5**). This triterpene degradation pathway was found to contribute to the formation of the pathogen-induced volatile terpenes in Arabidopsis (Sohrabi et al., 2015).

## Understanding *in Planta* Function of the P450 of the Triterpene Pathway

The biochemical function of majority of the steroidal scaffold-modifying plant P450s was substantiated based on characterization of the null mutants or gene over-expression lines of the model plant Arabidopsis. This was achievable due to the available genomic resources including functional mutants and efficient genome manipulation tools for Arabidopsis. This approach ascertained the general roles of CYP51G, CYP85A, CYP90B-D, CYP710A, CYP724A,B, and CYP734A across the plant kingdom for the metabolism of the primary sterols and steroid hormones (Kim et al., 2005; Morikawa et al., 2006; Ohnishi et al., 2006b; Sakamoto et al., 2006; Arnqvist et al., 2008). However, several steroidal compounds (e.g., steroidal saponins and steroidal alkaloids) and majority of the triterpene compounds are biosynthesised in species-specific manner (Thimmappa et al., 2014; Augustin et al., 2015; Miettinen et al., 2017). The biochemical functions of the P450s of these species-specific pathways were assigned mostly using heterologous expression host, including the model plants (Arabidopsis and tobacco), model microbe (yeast) and insect cell lines. Although biochemical characterization of about 60 P450s of the plant triterpene pathway (excluding steroidal scaffolds) has been completed following expression in heterologous host, only a handful of them were analyzed to determine the *in planta* function in triterpene biosynthesis (Carelli et al., 2011; Geisler et al., 2013; Moses et al., 2015b; Misra et al., 2017). The *in planta* functions of the triterpene-modifying P450s are highlighted in the following sections.

### Oat CYP51H10

A chemical mutagenesis approach in diploid oat species, *Avena strigosa* led to the identification of ten independent saponin-deficient (*sad*) mutants that either could not produce saponins in root or had reduced level (Papadopoulou et al., 1999). Saponin deficiency in *sad* mutant resulted in compromised disease resistance to a variety of root-infecting fungal pathogens. One of these oat mutants, i.e., *sad2* was mutated in *CYP51H10* gene (Qi et al., 2006). The physiological and biochemical analysis of *sad2* provided a conclusive evidence for the involvement of CYP51H10 in root avenacins biosynthesis (Qi et al., 2006; Geisler et al., 2013). *sad2* mutant accumulated high level of β-amyrin, confirming the biochemical function of CYP51H10 as β-amyrin-modifying enzyme (Qi et al., 2006; Kunii et al., 2012; Geisler et al., 2013). Moreover, the accumulation of abnormally high level of β-amyrin in *sad2* triggered a ‘superhairy’ root phenotype due to the high rate of transformation of epidermal cells into root hair cells as compared to non-hair cells (Kemen et al., 2014). This observation suggested an important role of β-amyrin in root development.

### Medicago CYP716A12 and CYP72A67

The roles of CYP716A12 and CYP72A67 in triterpene pathway were precisely determined based on in-depth analysis of *M. truncatula* mutant lines (Carelli et al., 2011; Biazzi et al., 2015). CYP716A12 and CYP72A67 loss-of-function mutants were developed following activation tagging and/or targeting induced local lesions in genomes (TILLING) approaches (Porceddu et al., 2008). CYP716A12 loss-of-function *iha* mutants could not produce hemolytic sapogenins (e.g., hederagenin, bayogenin, medicagenic, zanhic acid) with a C28 carboxylation (Carelli et al., 2011). This study confirmed the *in planta* function of CYP716A12 as a β-amyrin C28-oxidase. In accordance with the specific role of CYP716A12 in hemolytic saponin pathway, non-hemolytic saponins (e.g., soyasaponins) could be detected in *iha* mutants, similar to the wild type counterpart (Carelli et al., 2011). Interestingly, *iha* mutants showed severe growth retardation and altered gene expression related to the secondary metabolism and hormonal pathways, suggesting an important role of the hemolytic saponin biosynthesis pathway in plant growth processes (Carelli et al., 2011).

Moreover, genetic and biochemical analysis of the *CYP72A67* TILLING mutants provided substantial evidences for its specific role in hemolytic saponin pathway by catalyzing C2 oxidation of oleanolic acid/hederagenin (Biazzi et al., 2015). Hemolytic sapogenins with C2 hydroxylation (bayogenin, medicagenic, zanhic acid) were completely absent in *CYP72A67* mutants. However, sapogenins (gypsogenin, gypsogenic acid, and 16α-hydroxy gypsogenic acid) that lacked a C2 hydroxylation could be detected in *CYP72A67* mutants (Biazzi et al., 2015). Interestingly, an alteration in nodulation pattern was observed in *CYP72A67* mutant as compared with the wild-type plants, suggesting a potential role for the saponins in regulation of nodulation.

### Artemisia CYP716A14v2

Heterologous expression of *A. annua* CYP716A14v2 in yeast assigned its biochemical function as amyrin C3-oxidase. CYP716A14v2 converted α-amyrin, β-amyrin, and δ-amyrin to α-amyrone, β-amyrone, and δ-amyrone, respectively (Moses et al., 2015b). To access whether CYP716A14v2 catalyzes the same biochemical reaction *in planta*, transgenic *A. annua* plants were generated following RNA interference (RNAi) that resulted in silencing of the *CYP716A14v2* transcript expression (Moses et al., 2015b). The metabolite analysis of the RNAi and control plants revealed reduced levels of the α-amyrone and β-amyrone in *CYP716A14v2*-silenced plants as compared to the control, confirming the *in planta* role of CYP716A14v2 as amyrin C3-oxidase.

### Cucumber CYP88L2/CYP81Q58 and Sweet Basil CYP716A252/CYP716A253

The physiological function of cucumber (*C. sativus*) and sweet basil (*Ocimum basilicum*) P450s of the tetracyclic and pentacyclic triterpene pathways, respectively, was determined following transient gene silencing approaches. The *in planta* role of *CYP88L2* and *CYP81Q58* in cucurbitacin biosynthesis was probed by silencing their transcript expression in the cotyledons using a transient RNAi system. The down-regulation of *CYP88L2* and *CYP81Q58* transcripts resulted in decreased level of cucurbitacin in the cotyledons, confirming their involvement in cucurbitacin biosynthesis (Shang et al., 2014). Similarly, the *in planta* function of sweet basil CYP716A252 and CYP716A253 in the biosynthesis of the medicinally important pentacyclic triterpenes (ursolic acid and oleanolic acid) was clarified following a virus induced gene silencing (VIGS) approach (Misra et al., 2017). The down-regulation of *CYP716A252* and *CYP716A253* expression in sweet basil leaves resulted in reduced level of ursolic acid and oleanolic acid, suggesting that both of these amyrin C28-oxidases are required for the biosynthesis of ursolic acid and oleanolic acid in sweet basil leaves. However, a major contribution of the *CYP716A253* in elicitor-mediated accumulation of ursolic acid and oleanolic acid in sweet basil leaves was revealed. These studies in cucumber and sweet basil, suggested that transient gene silencing assays can be useful to probe the *in planta* function of P450s for the biosynthesis of the species-specific metabolites in non-model plants.

### Arabidopsis CYP71A16, CYP705A1, CYP705A5, and CYP708A2

The *in planta* function of two P450s (CYP705A5 and CYP708A2) of the Arabidopsis thalianol gene cluster was precisely determined following biochemical analysis of the RNAi or T-DNA insertion lines (Field and Osbourn, 2008). *CYP705A5* and *CYP708A2* co-expressed with the OSC *thalianol synthase* and catalyzed two consecutive biosynthetic steps of the thalianol pathway (**Figure 5**). *CYP708A2* mutant line lacked thalian-diol (7β-hydroxy-thalianol) and, however, accumulated increased level of thalianol (Field and Osbourn, 2008). These observations confirmed the physiological role of CYP708A2 as a thalianol hydroxylase. Similarly, metabolite analysis of the *CYP705A5* mutant lines revealed increased level of thalian-diol and absence of desaturated thalian-diol, confirming the *in planta* role of *CYP705A5* in thalian-diol desaturation. Interestingly, CYP708A2 over-expression line that accumulated higher level of thalian-diol had dwarf phenotype and longer roots than the wild type, suggesting a crucial role of thalian-diol in plant growth and development.

The physiological role of Arabidopsis CYP705A1 in volatile (E)-4,8-dimethyl-1,3,7-nonatriene (DMNT) production in roots has been well documented (Sohrabi et al., 2015). CYP705A1 catalyzed cleavage of the prenyl side chain of arabidiol to produce volatile DMNT and a non-volatile 14-apo-arabidiol (**Figure 5**). CYP705A1 and arabidiol synthase clustered in the Arabidopsis genome. These genes co-expressed in roots, and also responded to the jasmonic acid treatment and pathogen infection (Sohrabi et al., 2015). The gene knockout line *cyp705a1-1* could not emit DMNT and showed compromised defense toward the root-rot pathogen *Pythium irregulare*. Moreover, DMNT inhibited *in vitro* spore germination and growth of *P. irregulare*, suggesting that CYP705A1-mediated cleavage of arabidiol is an important defense mechanism of Arabidopsis.

The function of Arabidopsis CYP71A16 that constitutes the marneral cluster along with the OSC marneral synthase (MRN1) was also determined following biochemical analysis of the T-DNA mutant and over-expression lines (Field et al., 2011). Unlike the wild-type plants, *CYP71A16* T-DNA mutant lines accumulated marnerol, an alcohol spontaneously produced from marneral. Moreover, hydroxylated marnerol derivatives were detected in *CYP71A16* and *MRN1* over-expression lines (Field et al., 2011). These results clearly indicated the physiological role of *CYP71A16* in marneral/marnerol oxidation. Interestingly, Arabidopsis plants that over-expressed *CYP71A16* and *MRN1*, had pronounced dwarf phenotype, suggesting a detrimental effect of the marneral pathway intermediates on plant growth and development (Field et al., 2011).

## Conclusion

So far, about eighty P450s were assigned specific functions to the plant triterpene metabolism (including steroidal scaffolds). Considering a huge structural diversity of the plant triterpenes, it is quite obvious that several other P450s with uncharacteristic biochemical features are yet to be known. With the availability of the large scale genomic and transcriptomic sequence information (The 1000 plants ^1^; Medicinal Plant Genomics Resource ^2^), an increasing number of P450s are expected to be assigned function in the plant triterpene metabolism. The future challenges are functional characterization of the P450s having unusual biochemical properties, and utilization of the P450s for the plant improvement program and for the production of valuable phytochemicals.

Nevertheless, notable advances in understanding the roles of the P450s in plant triterpene metabolism for the general as well as species-specific functions have been made. Usually CYP51G, CYP85A, CYP90B-D, CYP710A, CYP724B, and CYP734A subfamily members are found to metabolize sterols and steroid hormones that mediate primary functions in plants. However, CYP51H, CYP71A,D CYP72A, CYP81Q, CYP87D, CYP88D,L, CYP93E, CYP705A, CYP708A, and CYP716A,C,E,S,U,Y subfamily members are generally found to metabolize triterpenes that showed species-specific distribution in plants. The physiological roles of the P450s in metabolism of the sterols and steroid hormones were mostly revealed based on gene function analysis in the model plants like Arabidopsis, tomato, and rice. Although, a number of P450s were identified from non-model plants and biochemically characterized following protein expression in heterologous hosts, including model plants (Arabidopsis and tobacco) and microbe (*S. cerevisiae*), the *in planta* function of a limited number of these P450s was determined. Therefore, extending the functional genomics tools (e.g., VIGS, RNAi, CRISPR/Cas9) to the non-model plants is crucial to know the biochemical function of the species-specific P450s and also to establish the biological roles of the triterpene specialized metabolites in plants.

## Author Contributions

SG collected literatures and wrote the manuscript.

## Conflict of Interest Statement

The author declares that the research was conducted in the absence of any commercial or financial relationships that could be construed as a potential conflict of interest.

## Footnotes

1 onekp.com/

2 medicinalplantgenomics.msu.edu/
